# New variants and genotype-phenotype correlation of *PPP3CA*-related developmental and epileptic encephalopathy

**DOI:** 10.3389/fnins.2025.1570997

**Published:** 2025-06-06

**Authors:** Ting Wang, Shijia Ouyang, Xueyang Niu, Miaomiao Cheng, Ying Yang, Yonghua Yang, Quanzhen Tan, Wenwei Liu, Xiaoling Yang, Yuehua Zhang

**Affiliations:** ^1^Children’s Medical Center of Peking University First Hospital, Beijing, China; ^2^Department of Respiratory, Beijing Children’s Hospital, Capital Medical University, Beijing, China; ^3^Department of Pediatrics, the First Affiliated Hospital of Xi’an Jiaotong University, Xi’an, China

**Keywords:** PPP3CA, variants, genotype, phenotype, developmental and epileptic encephalopathy

## Abstract

**Objective:**

To explore the genotypic spectrum and refine the genotype-phenotype correlation of *PPP3CA*-related developmental and epileptic encephalopathy (DEE).

**Methods:**

whole-exome sequencing or whole-genome sequencing was performed to all patients. Clinical data of 15 epilepsy patients in current study and 21 epilepsy patients from published studies were collected and analyzed.

**Results:**

In this study, 15 patients were identified with 13 *de novo PPP3CA* variants. Among these, seven frameshift variants and one gene inversion between intron 11 and intron 13 (including exons 12 and 13) were novel. 80% of patients experiencing seizure onset before the age of one. The seizure types observed included epileptic spasms (93.3%), tonic seizures (46.7%), myoclonic seizures (46.7%), focal seizures (40.0%), atypical absence seizures (13.3%), generalized tonic-clonic seizures (6.7%) and myoclonic atonic seizures (6.7%). All patients exhibited global developmental delay. MRI abnormalities were noticed in 9 patients, including widened subarachnoid space, bilateral ventricular width, poor myelination of white matter, and dysplasia of the corpus callosum. 80% specifically diagnosed with infantile epileptic spasms syndrome (IESS). When combining data from this study and published studies, 66.7% of patients experienced seizure onset before the age of one, and 77.8% were diagnosed with IESS. In patients with variants located in the catalytic domain (CD), 45.4% patients exhibited multiple seizure types, while 45.4% patients presented only with epileptic spasms. In contrast, among patients with variants in regulatory domain (RD), 87% had multiple seizure types and only 8.7% had epileptic spasms alone. Additionally, 45.5% of patients with CD variants had comorbid autism spectrum disorders, compared to 13% patients with RD variants. Recurrent variants included p.His92Arg, p.Asp234Glu, p.Glu282Lys, and p.Ser419Asnfs*31.

**Conclusion:**

This study is the first to report a gene inversion in *PPP3CA*-related DEE. Patients with only epileptic spasms were more prevalent in those with CD variants, compared to those with RD variants. Conversely, patients with multiple seizure types were more common among those with RD variants. The most frequently diagnosed epileptic syndrome was IESS. Additionally, comorbid ASD were more commonly observed in patients with CD variants than in those with RD variants.

## Introduction

Calcineurin, a calcium/calmodulin-regulated serine/threonine protein phosphatase, is a heterodimeric protein composed of two subunits: the catalytic A (CnA) and the regulatory B (CnB) (Aramburu et al., 2004; Creamer, 2020; Karagiota et al., 2019; Rusnak and Mertz, 2000). The *PPP3CA* gene (MIM:114105), located on chromosome 4q24, encodes the α-isoform of CnA and mediates Ca^2+^-dependent signal transduction (Cook and Creamer, 2016; Creamer, 2013; Palkowitsch et al., 2011). It is widely distributed in the mammalian brain, particularly enriched in synapses, and is involved in the recycling of synaptic vesicles at nerve terminals (Dhindsa et al., 2015). *PPP3CA* consists of five domains: the catalytic domain (CD), the calcineurin regulatory subunit-calcineurin binding domain (CnBB), the regulatory domain (RD), the calmodulin binding domain (CaMB) and the auto-inhibitory domain (AID) (Li et al., 2016; Rumi-Masante et al., 2012).

In 2017, Myers et al. (2017) first reported that the variants of *PPP3CA* are associated with developmental and epileptic encephalopathy (DEE). In 2018, Mizuguchi et al. (2018) identified six heterozygous *PPP3CA* variants in four patients with DEE and two patients with multiple congenital malformation. Using a yeast model, they identified two functionally distinct types of variants: loss-of-function variants, characterized by decreased calcineurin signaling at the CD, and constitutively activating variants, characterized by increased calcineurin signaling at the AID. Panneerselvam et al. (2021) suggested that *PPP3CA* truncating variants cluster in the RD domain, leading to more severe early-onset refractory epilepsy. The present study identified novel variants and analyzed the genotype-phenotype correlation in conjunction with published literature.

## Materials and methods

### Patients

Genetic testing was conducted on all patients diagnosed with epilepsy without acquired factors such as perinatal brain injury, traumatic brain injury, central nervous system infections, etc. From August 2019 to July 2024, a total of 3350 cases of children with epilepsy and single gene variants were enrolled. Among them, 0.45% (15/3350) were found to have *PPP3CA* heterozygous variants. This study summarized the patients’ seizure onset age, seizure types, developmental milestones, neurological status, family history, and results of ancillary examinations, including electroencephalogram (EEG) and brain magnetic resonance imaging (MRI). The normal value of the extracranial space is based on reference (McArdle et al., 1987; Wang et al., 1996b), and the expansion of the extracranial space is defined as exceeding the average value of the corresponding age group plus 2 times the standard deviation. The determination of myelination process can be found in reference (Wang et al., 1996a). Treatment and prognosis were also followed up in the clinic.

This study was approved by the Ethics Committee of Peking University First Hospital [Approval number 2012 (453)]. The children with epilepsy and *PPP3CA* variants included in this study were diagnosed according to the 2017 epilepsy classification by the International League Against Epilepsy (ILAE) (Fisher et al., 2017) and 2022 epilepsy syndrome classification of ILAE (Zuberi et al., 2022). All procedures involving human participants in this study adhered to the 1964 Declaration of Helsinki and its later amendments or similar ethical standards. Parental written informed consent was obtained from each patient’s guardian.

### Genetic testing

Variant screening of *PPP3CA* (NM-000944.4) was conducted using next-generation sequencing. Fourteen patients were screened using whole-exome sequencing (WES), while one patient was screened using whole-genome sequencing (WGS). High-throughput sequencing was performed using the Agilent Sure Select Method Exome V6 on the Illumina sequencing platform. The sequencing data were aligned and analyzed using NextGENe^®^ software, and variants were screened and interpreted using the Ingenuity online software system. Gene-specific testing was performed on the parental DNA samples for all *PPP3CA* variants and on the siblings of the probands for segregation analysis. We have identified the allele frequencies for various genetic variants utilizing data from the Genome Aggregation Database (gnomAD).^1^ The pathogenicity of variant was assessed according to the guidelines of the American College of Medical Genetics and Genomics (ACMG) (Chen et al., 2018; Richards et al., 2015).

The DNA of the patient 5 and his parents were analyzed using the Qsep100 fully automatic nucleic acid analysis system (BioNer Inc., Korea) (Gan et al., 2014; Zhuang et al., 2015). The Qsep100 system quantifies DNA fragments through fluorescence detection and simulates the band pattern as discrete peaks. The target gene was amplified by the primers listed below and then capillary electrophoresis was performed on the Qsep100 system. The detailed procedure could be obtained in Supplementary Data Sheet 1. The PCR conditions and primer specificity were verified through chip analysis (Primer-blast) and empirical optimization. The sequence of PCR primers was as follows,

*PPP3CA*-1F GCATTTCTGTTTTGTTGTCTGT,

*PPP3CA*-1R CATCATATACAAAAGTCAAACATAGCT;

*PPP3CA*-2F CACAGAAAAGAGTTGCATTTGTATT,

*PPP3CA*-2R TCACATCAACTGCTTATTTTAATGTC.

### Protein modeling and analysis

The *PPP3CA* protein sequences were obtained from NCBI. We utilized AlphaFold2 (V.2.3.2) for the monomer’s prediction (Jumper et al., 2021). The prediction models were evaluated based on each residue’s confidence score and were ranked according to their confidence level, with 0 representing the output file with the highest confidence. File with a residual confidence of 0 was selected as the final prediction for further analysis (pTM = 0.82). AlphaFold2 prediction result was shown in Supplementary Figure S1. The open-source Pymol software (V.2.5, by Schrödinger, New York, United States) was utilized to visualize the three-dimensional protein structure, facilitating the analysis of the variants’ position within the *PPP3CA* domain.

### Literature review

Literature with “*PPP3CA*” and “Epilepsy” as keywords, published in PubMed and Web of Science from October 2017 to October 2024, was searched. A total of 21 patients with *PPP3CA* variants were identified across 8 studies. Supplementary Table S1 summarized the age of seizure onset, seizure type, patient development, EEG, brain MRI and other clinical data from the selected reports (Li and Cao, 2022; Mizuguchi et al., 2018; Myers et al., 2017; Panneerselvam et al., 2021; Qian et al., 2018; Rydzanicz et al., 2019; Yang et al., 2020).

### Statistical analysis

Statistical analysis was performed using GraphPad Prism 8. Data were analyzed using Fisher’s exact tests for significance at the bivariate level. Exact *p*-values are reported, with significance defined as *p* < 0.05.

## Results

In total, 15 patients with *PPP3CA* variants were identified, and their genotype and clinical features are shown in Table 1. This study included 11 males and 4 females, with the last follow-up age ranging from 7 months to 6 years.

**TABLE 1:** The genotype and phenotype of 15 patients with *de novo PPP3CA* variants in this study.

	Sex	Age of last evaluation	Variants	Inheritance	Location	Seizure		Develop-ment	Other clinical findings	EEG	Brain MRI	Diagnosis	ASMs
						**Age of onset**	**Seizure type**						
1	F	5y1m	c.275A>G/ p.His92Arg	*De novo*	CD	2y9m	ES	DD	Hypotonia	Spike wave, spike slow wave, multiple spike slow complex and slow wave	N	IESS	VPA, CZP, ACTH, LEV, VGB
2	M	2y2m	c.702C > A/ p.Asp234Glu	*De novo*	CD	6m	ES, MS	DD	Microcephaly, hypospadias, ASD	Multifocal discharges and hypsarrhythmia	Subarachnoid space Wide, white matter dysplasia	IESS	**TPM, LTG, VGB,** ACTH, VitB6
3	M	1y6m	c.275A>G/ p.His92Arg	*De novo*	CD	6m	ES	DD	N	Multifocal discharges and hypsarrhythmia	N	IESS	**TPM, VGB,** ACTH, VitB6
4	M	11m	c.702C > A/ p.Asp234Glu	*De novo*	CD	3m	ES	DD	Hypotonia	Hypsarrhythmia	N	IESS	**TPM, VGB,** ACTH
5	M	4y5m	inversion between intron 11-13	*De novo*	RD	1y8m	GTCS, MS, ES, FS, Ats	DD	Hypotonia, Microcephaly	Generalized spike, spike slow wave, sharp slow wave	Subarachnoid space wide	DEE	**VPA, LEV,** TPM, PB, **CZP**
6	M	3y	c.1255_1256delAG/ p.Ser419Cysfs*31	*De novo*	RD	9d	T, ES, MS, MAS	DD	Hypotonia	Hypsarrhythmia	N	IESS	**VPA, LTG, VGB,** ACTH
7	F	4y11m	c.1284_1287dup/ p.Gly430Asnfs*22	*De novo*	RD	1y3m	MS	DD	N	2–3 Hz spike wave and s pike slow wave	N	DEE	**VPA, LEV, CZP,** LTG, NIT
8	M	4y	c.1336_1337insCAAA/ p.Ser446Thrfs*6	*De novo*	RD	2m	FS, ES	DD	Hypertonia	Hypsarrhythmia	Dysplasia of corpus callosum	IESS	**VPA, TPM, VGB, CZP, LEV,** ACTH
9	M	3y6m	c.1338dup/p. Ala447fs* 4	*De novo*	RD	9m	ES, T, FS	DD	N	Atypical hypsarrhythmia	Subarachnoid space wide, white matter dysplasia	IESS	**VPA, TPM, VGB,** ACTH
10	M	3y	c.1258_1259 insAGTG/ p.Vla420Glufs*32	*De novo*	RD	4m	ES, T	DD	Hypertonia	Burst suppression	Subarachnoid space wide	IESS	**VPA, LEV**, ACTH, CZP, LAM, VGB, KD
11	M	5y5m	c.1283_1284insC/ p.Thr429Asnfs*22	*De novo*	RD	2y	ES, TS, MS, Ats	DD	Hypertonia	Generalized epileptiform discharges, atypical hypsarrhythmia	N	IESS	**VPA, CLB, LTG,** ACTH, LEV, VGB, KD
12	M	2y5m	c.1311_1312insACTT/ p.Ser438fs*14	*De novo*	RD	4m	ES	DD	Hypotonia, Microcephaly, ASD, difficulty in feeding	Hypsarrhythmia, suppression-burst	Subarachnoid space wide, white matter dysplasia, dysplasia of corpus callosum	IESS	VGB, VPA, ACTH
13	M	1y8m	c.1354_1356delins CAATA/ p.Ile452Glnfs*2	*De novo*	RD	2m	ES, FS, T, MS	DD	Hypotonia, gingival hyperplasia, Wide teeth,	Multifocal discharges and hypsarrhythmia	Corpus callosum dysplasia	DEE	**VPA, LTG, PER**, ACTH, TPM, VGB, KD
14	M	1y3m	c.375A > T/ p.Lys459*	*De novo*	RD	2m	ES, FS, TS, MS	DD	Hypotonia, arthrogryposis	Background slow wave, hypsarrhythmia	Ventricular width bilaterally, agenesis of corpus callosum	IESS	**TPM, LEV, OXC,** VGB**, CZP,** ACTH, PB
15	F	7m	c.1251_1252del/ p.Ser417Argfs*33	*De novo*	RD	3m	ES, FS, TS	DD	Hypertonia	Hypsarrhythmia	Subarachnoid space wide, ventricular width bilaterally	IESS	LEV, **VPA CLB, TPM**, VGB, ACTH

M, male; F, female; d, days; m, months; y, years; RD, Regulatory domain; CD, catalytic domain; Ats, atypical absence seizures; ES, epileptic spasm; MS, myoclonic seizure; FS, focal seizure; GTCS, generalized tonic–clonic seizure; T, tonic seizure; DEE, developmental and epileptic encephalopathy; DD, developmental delay; ASD, Autism Spectrum Disorder; IESS, infantile epileptic spasms syndrome; EEG, electroencephalography; MRI, magnetic resonance imaging; N, normal; ACTH, adrenocorticotropic hormone; ASMs, Anti-Seizure Medications; LEV, levetiracetam; VPA, valproic acid; LAM, lamotrigine; TPM, topiramate; CLB, clobazam; VGB, vigabatrin; PB, phenobarbital; NIT, nitrazepam; PER, perampanel; CZP, clonazepam; KD, ketogenic diet; LTG, lamotrigine; VitB6, vitamin B6; OXC, oxcarbazepine.

### Genetic analysis

**FIGURE 1:**
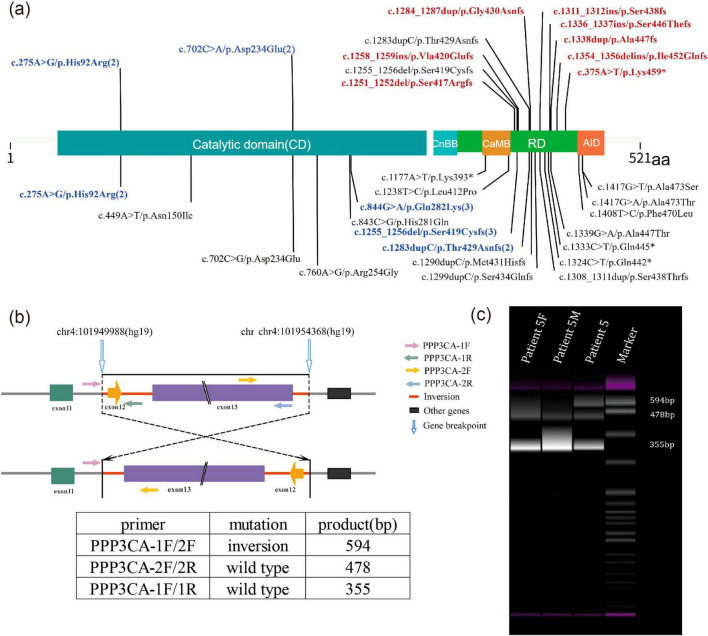
**(a)** Schematic representation of the location of *PPP3CA* (NM_000944.4) variants in this study and in existing literature. Variants previously described in the literature are shown at the bottom of the diagram, while those found in this study are shown at the top. Novel variants reported for the first time in this study are indicated in red. Sites with more than 2 variants are indicated in blue. *PPP3CA* contains five domains: the catalytic domain (CD), the calcineurin regulatory subunit-calcineurin binding domain (CnBB), the regulatory domain (RD), the calmodulin binding domain (CaMB) and the auto-inhibitory domain (AID). **(b)** Schematic representation of the inversion and primer design for Patient 5. The inversion region is located between introns 11 and 13, including exons 12 and 13. **(c)** PCR results showing an abnormal product of 594 bp. Patient 5F, DNA sample of patient 5’s father; Patient 5M, DNA sample of patient 5’s mother.

Pathogenic or likely pathogenic of *PPP3CA* variants were identified in 15 children with epilepsy. The ACMG score results of these variants can be found in Supplementary Table S2. Thirteen unique *de novo* variants were detected, including 10 frameshift variants, 2 missense variants, and 1 gene inversion located between intron 11 and 13. Two recurrent variants were identified: His92Arg and Asp234Glu, as shown at the top of the Figure 1a and in Table 1. To predict the potential impact of missense mutations on the biological functions of proteins, we use Mutation Taster, polyphen2 and SIFT, etc. to predict pathogenicity. The specific results are shown in Supplementary Table S3 and Supplementary Figure S2. Seven truncating variants (indicated in red in Figure 1a) were reported for the first time, namely p.Gly430Asnfs*22, p.Ser446Thrfs*6, p. Ser438fs*14, p. Ala447fs*4, p.Ile452Glnfs*2, p.Lys459* and p.Ser417Argfs*33. These variants were absent from population frequencydatabases (gnmAD v3.1.2).

A unique *de novo* gene inversion was identified in Patient 5, spanning from intron 1l to intron 13 (encompassing exon 12 and exon 13). This inversion was detected through whole-genome sequencing (WGS). Notably, as exon 13 represents the terminal exon of this gene, this structural alteration does not lead to novel protein production from subsequent genetic elements. Furthermore, the mutation does not exert any functional impact on downstream genes through positional effects. The subsequent genes can be queried through NCBI (NC_000004.12). Subsequent PCR validation in the patient revealed an additional abnormal product of 594 bp. In contrast, the normal parental samples exhibited products of 478 and 355 bp (Figures 1b,c).

### Variants’ location in protein structure

**FIGURE 2:**
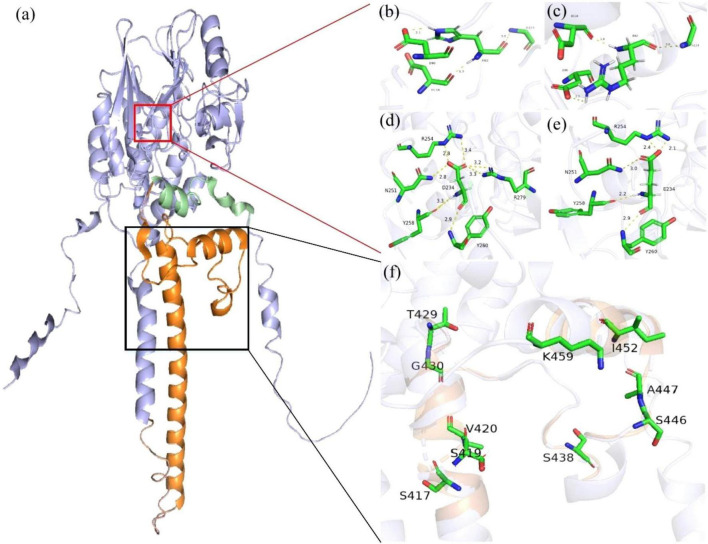
Protein structure of human *PPP3CA* (Q08209). **(a)** The regulatory domain (RD) is shown in orange, the auto-inhibitory domain (AID) in green, and the catalytic domain (CD) in purple. **(b–e)** In the variant model, the hydrogen bonds changed a lot, and the original hydrogen bonds broke, forming new hydrogen bonds. Hydrogen bonds of residues interacting with other amino acids (yellow) and the number represents the direct distance of the hydrogen bond. **(f)** The detailed structure of the black box highlights the other nine truncating variants that occur in the RD.

Figure 2a displays the 3D structural modeling of *PPP3CA* variants, with 12 identified variants clustering in two distinct regions. The p.Asp234Glu and p.His92Arg variants localize to the catalytic domain’s active site (Figure 2a), both showing reduced hydrogen bonding and altered interactions with surrounding residues (Figures 2b–e). Nine truncating variants occurred in the regulatory domain (Figure 2f).

### Clinical phenotypes

In our study of 15 epileptic patients carrying *PPP3CA* variants, seizure onset occurred from 9 days to 2 years and 9 months, with 80% (12/15) experiencing seizure onset before the age of one. Epileptic spasms were the predominant seizure type, affecting 93.3% (14/15) of patients. Other seizure types included tonic seizures in 46.7% (7/15), myoclonic seizures in 46.7% (7/15), focal seizures in 40.0% (6/15), atypical absence seizures in 13.3% (2/15), generalized tonic and clonic seizures (GTCS) in 6.7% (1/15), and myoclonic atonic seizures in 6.7% (1/15). Four patients presented only with epileptic spasms, three of whom (patient 1, 3, 4) had variants located in the CD, and one patient (patient 7) presented only with myoclonus.

In this study, all patients exhibited developmental delay and intellectual disability. Twelve patients were unable to walk after the age of 1 year and 6 months. Among those over 2 years old, nine were still unable to walk and speak. Five patients over 3 years old remained unable to walk and speak. Patient 7 was able to walk independently at 13 months and could say limited words by the age of 4 years and 11 months at the last follow-up. Additionally, 10 patients manifested hypotonia, three had microcephaly, two experienced feeding difficulties, one had gingival hyperplasia, and one had hypospadias. Patient 2 was diagnosed with autism spectrum disorder (ASD). The clinical features are summarized in Table 1.

### Video EEG and brain imaging

**FIGURE 3:**
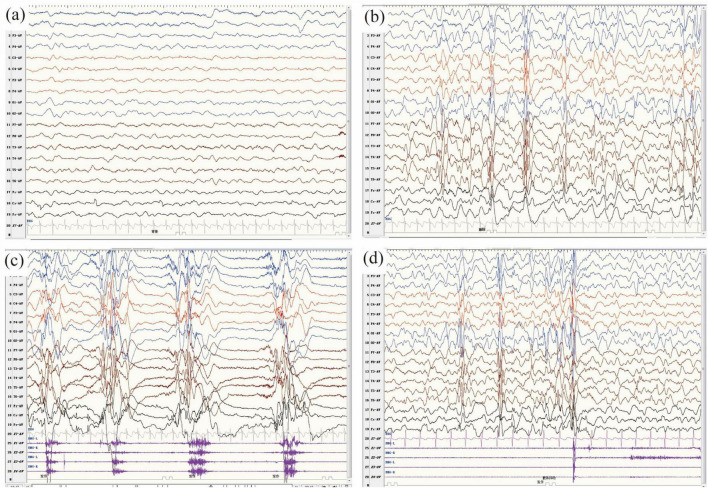
**(a–d)** Video electroencephalography (VEEG) monitoring of patient 11 in this study at the age of 10 months. **(a)** The background activity was slow. **(b–d)** The interictal VEEG demonstrated hypsarrhythmia, characterized by numerous generalized and multifocal spikes, as well as multiple spikes and slow waves, which occurred intermittently or continuously during both waking and sleeping periods. **(c,d)** The ictal VEEG captured clusters of epileptic spasms **(c)** and myoclonic seizures **(d)**.

Fifteen patients underwent video EEG for 4–24 h on multiple occasions (Table 1). The VEEG revealed diffuse slow background activity in three patients, hypsarrhythmia in 12 patients, burst suppression in two patients, generalized discharges in three patients, and multifocal discharges in three patients. Epileptic spasms were detected in 13 patients, tonic seizures in five patients, myoclonic seizures in four patients, atypical absence seizures in two patients, tonic-spasms in two patients, and GTCS in one patient. The video EEG of Patient two is shown in Figure 3. His video EEG displayed a slow background. The interictal EEG showed hypsarrhythmia, with numerous generalized and multifocal spikes, as well as multiple spikes and slow waves during both waking and sleeping periods. Epileptic spasms and myoclonic seizures were detected.

**FIGURE 4:**
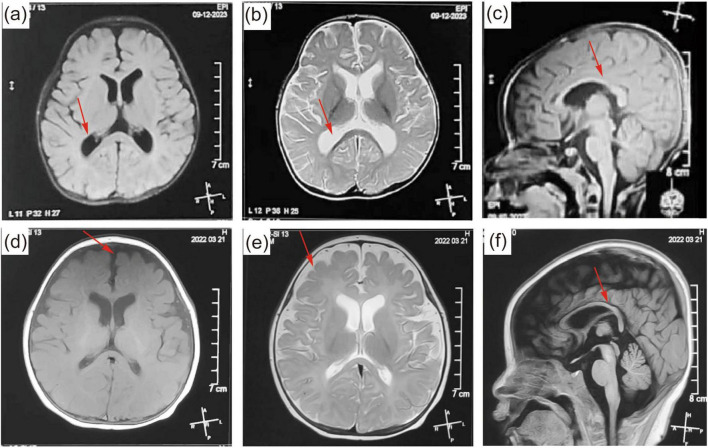
**(a–c)** Brain magnetic resonance imaging (MRI) of patient 9 at the age of 6 months. The brain MRI showed a widened subarachnoid space, white matter dysplasia, and dysplasia of corpus callosum. **(d–f)** MRI of patient 13 at the age of 7 months. The brain MRI showed enlarged bilateral ventricles and agenesis of the corpus callosum. The abnormal parts in the brain MRI are pointed out by red arrows.

Fifteen patients underwent brain MRI. Abnormalities were detected in the brain of nine patients, including subarachnoid space wide in six patients, corpus callosum dysplasia in three patients, white matter dysplasia in three patients, and enlarged bilateral ventricles in two patients. The brain MRI of patient 4 is shown in Figures 4a–c. His brain MRI showed enlarged bilateral ventricles and agenesis of the corpus callosum. The brain MRI of patient 12 is shown in Figures 4d–f. His brain MRI revealed a widened subarachnoid space, white matter dysplasia, and dysplasia of corpus callosum.

### The diagnosis of epileptic syndrome

In this study, 11 patients were diagnosed with infantile epileptic spasms syndrome (IESS). The remaining three patients were diagnosed with developmental and epileptic encephalopathy (DEE), which could not be further classified into a specific epileptic syndrome.

### Literature review and analysis

As of October 2024, 26 patients with *PPP3CA* variants have been reported in the literature (Jumper et al., 2021; Li and Cao, 2022; Li et al., 2016; Mizuguchi et al., 2018; Myers et al., 2017; Qian et al., 2018; Rydzanicz et al., 2019; Yang et al., 2020). Three patients with variants in the AID exhibited only developmental delay and facial dysmorphic features, and were therefore excluded (Mizuguchi et al., 2018; Panneerselvam et al., 2021). Additionally, two patients (p.Glu282Lys in CD) with ASD but without seizures were excluded (Myers et al., 2017; Panneerselvam et al., 2021). The remaining 21 epileptic patients with variants in the CD, CaMB, and RD were described with detailed clinical manifestations (Table S1). Among these, eight were missense variants (p.His92Arg, p.Asn150Ile, p.Asp234Glu, p.Arg254Gly, p.His281Gln, p.Glu282Lys, p.Leu412Pro, p.Ala447Thr), six were frameshift variants (p.Ser419Cysfs* 31, p.Val420Glufs 32, p.Thr429Asnfs*22, p.Met431Hisfs*20, p.Ser434Glnfs*17, p.Ser438Thrfs*14), and three were nonsense variants (p.Lys393*, p.Gln442*, p.Gln445*). Three variants p.His92Arg (*n* = 4), p.Glu282Lys (*n* = 3) and p.Ser419Cysfs* 31 (*n* = 3) were reported from more than one patient.

In 21 patients with *PPP3CA*-related epilepsy reported in the literature, the age of seizure onset ranged from 1.5 months to 13 years, with 57.1% (12/21) experiencing their first seizure before the age of one. Multiple seizure types were documented, including epileptic spasms (*n* = 16), myoclonic seizures (*n* = 9), tonic seizures (*n* = 8), generalized tonic-clonic seizures (*n* = 7), focal seizures (*n* = 7), atypical absence seizures (*n* = 5), typical absence seizure (*n* = 1), and clonic absence (*n* = 1). Two or more seizure types were identified in 17 patients. All patients exhibited developmental delay, and seven were diagnosed with autism spectrum disorder (ASD). Hypotonia was observed in 73.9% (17/23) of patients.

The VEEG exhibited diffuse slow background activity in 4 patients, hypsarrhythmia in 12 patients, burst suppression in 2 patients, generalized discharges in 2 patients, and multifocal discharges in 9 patients.

All 21 patients underwent brain MRI. Nine patients showed abnormalities, with the main manifestations being generalized prominence of subarachnoid spaces, brain dysplasia, thin corpus callosum, and widened brain interval. Twelve patients had normal brain MRI results, The MRI features of 21 patients were summarized in Supplementary Table S1.

### Genotype–phenotype correlation

Genotype-phenotype correlation analysis was performed in 36 patients with *PPP3CA* variants, including 15 from this study and 21 from published literature. The variants were classified and compared based on their location within different domains (Figure 1a). Eleven patients carried 8 variants in the CD, 2 patients carried 2 variants in the CaMB, and 23 patients carried 17 variants in the RD. Over 60% of the epilepsy-related variants were located in RD, which was more than any other domain. Variants in the CD were exclusively missense variants. In the CaMB, both missense and truncating variants were identified, and 94.1% (16/17) of the variants in the RD were truncating variants, including frameshift variants, nonsense variants and gene inversion.

**FIGURE 5:**
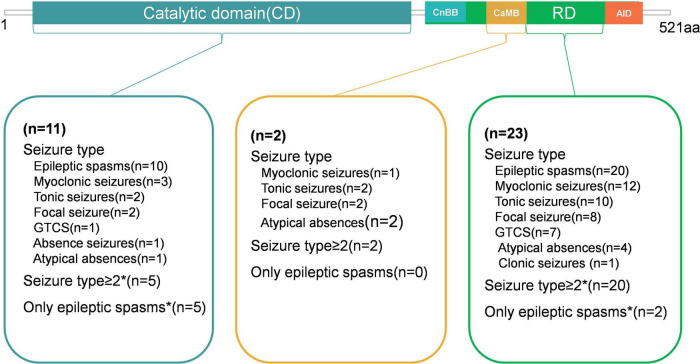
Diagram showing the Percentage of different seizure types in CD, CaMB and RD.

Figure 5 is the diagram showing the percentage of different seizure types in CD, CaMB and RD. In the CD, 63.6% (7/11) of patients experienced seizures within the first year. 45.4% (5/11) had multiple seizure types, while 45.4% (5/11) presented only with epileptic spasms. 72.7% (8/11) were diagnosed with IESS. MRI results were normal in 8 patients and abnormal in the remaining 3 (27.3%). 54.5% (6/11) of patients had ASD. The variant His92Arg was identified in 4 patients (11.1%), while Asp234Glu and Glu282Lys were found in 3 patients each (8.3%).

For patients with variants in the CaMB, the age of seizure onset ranged from 3 to 4 years. Both patients exhibited multiple seizure types, and MRI results were normal. In contrast, among patients with variants in the RD, 69.6% (16/23) experienced seizure onset before the age of one. 87% (20/23) had multiple seizure types, while 8.7% (2/23) patients presented only with epileptic spasms. 65.2% (15/23) patients had abnormal MRI results. The variant p.Ser419Cysfs*31 in RD was found in 4 patients (11.1%), and three patients presented epilepsy with ASD.

A comparison of patients with variations in CD and RD revealed. Patients with variants in the RD had a smaller mean age of seizure onset, although this difference was not statistically significant (*p* > 0.05). Compared to patients with variants in the CD, a significantly higher proportion of patients with variants in the RD had multiple seizure types (*p* < 0.05), while a significantly lower proportion in the RD presented only with epileptic spasms (*p* < 0.05). Patients with variants in the CD were more likely to have ASD compared to those with variants in the RD (*p* < 0.05). The variants. p.His92Arg, p.Asp234Glu Glu282Lys, and p.Ser419Cysfs*31were common variants among patients with *PPP3CA*-related epilepsy. These features are summarized in Table 2.

**TABLE 2:** Summary of clinical and epilepsy characteristics of 34 patients.

Clinical characteristic	Missense variants in CD (*n* = 11)	Truncating and inversion variants in RD (*n* = 23)	*P*-value
Sex (M/F)	4/7	16/7	0.1345
Age of seizure onset < 1 year	7/11 (63.6%)	16/23 (69.6%)	> 0.05
**Seizure type**			
*ES*	10/11 (90.9%)	20/23 (87.0%)	/
*MS*	3/11 (18.2%)	12/23 (52.2%)	0.2714
*TS*	2/11 (18.2%)	10/23 (42.9%)	0.2525
*GTCS*	1/11 (9.1%)	7/23 (30.4%)	0.2275
Seizure type ≥ 2*	5/11(45.5%)	20/23(87.0%)	0.0328
Developmental disability	11/11 (100%)	23/23 (100%)	/
Autism spectrum disorders*	6/11 (54.5%)	3/23 (13%)	0.0328
Abnormal EEG	11/11 (100%)	23/23 (100%)	/
Brain MRI abnormalities	3/11 (27.3%)	15/23 (65.2%)	0.0663

M, male; F, female; CD, catalytic domain; RD, Regulatory domain; ES, epileptic spasm; MS, myoclonic seizure; T, tonic seizure; DEE, developmental and epileptic encephalopathy; MRI, magnetic resonance imaging.

*p < 0.05.

### Epilepsy treatment and follow-up

The last follow-up age of our patients ranged from 7 months to 6 years. There was no significant seizure reduction in patients after two or more anti-seizure medications (ASMs) treatment. Thirteen patients with epileptic spasm were treated with adrenocorticotropic hormone (ACTH). For patient 4, epileptic spasms were controlled after ACTH treatment, but focal seizures still occurred intermittently. In patient 7 and 8, epileptic spasms were reduced but not fully controlled. The other 10 patients did not respond to ACTH treatment. Vigabatrin was administered to 13 patients with epileptic spasm, but it showed no reduction in seizures. Three patients were treated with the ketogenic diet for over 3 months, yet their seizures did not decrease, and their cognitive level did not improve significantly. Patient 10 had four seizure types, including epileptic spasm, myoclonic seizure, focal seizure and tonic seizure. His seizures were significantly reduced with valproic acid and lamotrigine, and he became seizure-free after receiving valproic acid, lamotrigine and vagus nerve stimulation (VNS) for 6 months before seizure recurred. Currently, he has 5-6 focal seizures per day. Patient 3, who had only myoclonic seizures was treated with valproic acid, levetiracetam and clonazepam, resulting in a 75% reduction in seizure frequency. At 4 years and 11 months, she could speak simple words and understand simple commands. Nine patients still could not sit unaided and had no language development after the age of 2. Cognitive regression was observed in three patients (patient 6, 8, 12) following seizure onset. The remaining three patients were under 1 years old, all showed unstable head control, lack of visual tracking, and absence of auditory responses.

## Discussion

Genetic etiology is increasingly recognized as a significant factor in epilepsy etiologies. Li et al. (2016) reported the first case of a patient with a *PPP3CA* variant in 2017. As of October 2024, a total of 26 patients with *PPP3CA* variants have been reported (Favaro et al., 2024; Li and Cao, 2022; Mizuguchi et al., 2018; Myers et al., 2017; Panneerselvam et al., 2021; Qian et al., 2018; Rydzanicz et al., 2019; Yang et al., 2020). Notably, previous studies have shown that pathogenic variants of *PPP3CA* are concentrated in four domains. These pathogenic variants can be categorized into two types based on clinical phenotype. Variants in the CD, CaMB, and RD result in the developmental and epileptic encephalopathy 91 (DEE91, MIM: 617711). DEE91 is characterized by epileptic spasms accompanied by global developmental delay or regression with intellectual disability, language impairment, and ASD in some patients. However, variants in AID lead to arthrogryposis, cleft palate, craniosynostosis, and impaired intellectual development (ACCIID; MIM: 618265) (Mizuguchi et al., 2018; Panneerselvam et al., 2021).

All *PPP3CA* variants identified in the patients are *de novo*, including missense variants, frameshift variants and nonsense variants. Six variants located in the CD were exclusively missense variants. Missense variants in the CD lead to reduced CaN signaling in yeast model, suggesting a loss-of-function mechanism (Mizuguchi et al., 2018). Two variants located in the CaMB were one missense variant and one nonsense variant (Favaro et al., 2024). However, the variants in the RD were predominantly truncating variants, with only one missense variant (Ala447Thr) reported. This missense variant is located at the last nucleotide of exon 12 of *PPP3CA*. Multiple splicing prediction programs suggested that this change would affect mRNA splicing sites, leading out-of-frame deletion of exon 12 due to exon skipping (Panneerselvam et al., 2021). Some studies have found that nonsense variants mediated mRNA decay, resulting in haploinsufficiency (Mizuguchi et al., 2018). The variant of AID domain disrupts the interaction with the catalytic domain, leading to the failure of self-suppression and thus resulting in gain-of-function (Mizuguchi et al., 2018). Other studies have shown that frameshift variants did not decrease mRNA expression levels but caused protein instability leading to haploinsufficiency (Qian et al., 2018; Rydzanicz et al., 2019).

In this study, all 15 patients had *de novo* variants, including 2 missense variants, 10 frameshift variants, and 1 gene inversion. The missense variant was located in the CD, consistent with previous literature. AlphaFold2 prediction revealed that the mutated amino acid had reduced interaction with surrounding amino acid residues, indicating a loss-of-function change. However, frameshift variants and gene inversions were located in the RD. A novel variant, gene inversion, was identified for the first time in our study. In addition, seven other novel frameshift variants were identified, further expanding the *PPP3CA* gene variation spectrum. Currently, truncating variants, including nonsense and frameshift variants, are the most common type of *PPP3CA* variants in the RD. There are few studies on truncating variants in *PPP3CA*, so the underlying mechanisms remain unclear.

Combining the patients with epilepsy from this study and those in the literature review, variants were identified in 13 patients in the CD, 2 patients in the CaMB domain and 23 patients in the RD. The variants p.His92Arg, p.Asp234Glu, p.Glu282Lys and p.Ser419Cysfs*33 were recurrent in *PPP3CA*.

In our study, 80% of patients experienced their first seizure onset before the age of one. In the literature review, among 21 patients with epilepsy, 57.1% had their first seizure before the age of one. Combining the data from our study and the literature review, 66.7% (24/36) of patients had their first seizure within their first year of life. In our study, multiple seizure types were observed, including epileptic spasms, tonic seizures, myoclonic seizures, focal seizures, GTCS, absence seizures, and myoclonic atonic epilepsy. Among the 21 patients reported previously, seizure types included epileptic spasms (16/21), myoclonic seizure (9/21), GTCS (9/21), focal seizure (7/21), tonic (4/21), atypical absence seizure (2/21), and absence seizure (1/21). Combining the results from our study and the literature, the most common seizure type in patients with *PPP3CA* variants was epileptic spasms (30/36, 83.3%).

In the literature review, epileptic syndromes were diagnosed in approximately 75% (16/21) of patients, which include IESS (*n* = 14), and Lennox–Gastaut syndrome (LGS, *n* = 2). In our study, 80% of patients were diagnosed with IESS. This suggests that IESS is the most common phenotype of *PPP3CA*-related epilepsy.

In the literature review, interictal EEG showed hypsarrhythmia, burst suppression, generalized discharges, and multifocal discharges. Epileptic spasms, focal seizures, myoclonic seizures, generalized tonic–clonic seizures, and absence seizures were observed in some patients (Panneerselvam et al., 2021). In this study, hypsarrhythmia was observed in 80% of patients, multifocal epileptiform discharges in 23.4%, generalized epileptiform discharges in 23.4%, and burst suppression in 13.3%. Epileptic spasms were detected in 13 patients, with patient 2 developing epileptic spasms for the first time nearly 3 years old. These findings suggest that hypsarrhythmia may be a characteristic EEG feature in the majority of patients with *PPP3CA*-related epilepsy. Among the 21 patients reviewed in the literature, about 50% had abnormal brain MRI, most commonly showing cerebral dysplasia and thin corpus callosum (Li and Cao, 2022; Li et al., 2016; Mizuguchi et al., 2018; Myers et al., 2017; Rydzanicz et al., 2019; Yang et al., 2020). In this study, abnormal brain MRI was observed in 10 patients, including frontotemporal subarachnoid widening, corpus callosum dysplasia, and white matter dysplasia.

Comparison of clinical features among patients with variants located in the CD, RD reveals distinct patterns. Patients with variants in the CD tend to have a relatively late seizure onset age, with nearly half patienting only had epileptic spasm. Additionally, nearly 50% of these patients have comorbid ASD, suggesting that ASD-related examinations should be recommended for patients with variant in the CD. However, patients with variants in the RD experienced an earlier seizure onset age. Approximately 90% of patients with RD variants had multiple seizure types, and about 75% exhibited brain MRI abnormalities, highlighting the importance of enhancing brain MRI for the diagnosis of *PPP3CA*-related DEE.

In the literature review, 33.3% (7/21) of the patients had epilepsy with ASD, and five of these had variants in the CD between Asn150 and Gln282 site. Additionally, two patients with p.Glu282Lys variants in the CD displayed ASD without seizures. In this study, patient 11, with variants located in CD, exhibited autism-like symptoms. This finding suggests that patients with variants located between Asn150 and Gln282 sites are more likely to have epilepsy with ASD. However, the mechanism of *PPP3CA* variants causing ASD requires further investigation.

Therapeutic information for *PPP3CA* related epilepsy is limited. Patients with this condition generally do not respond well to ASMs. In the literature review, one patient with the variant p.Glu282lys experienced seizure onset at 4 years old and became seizure free after the treatment with VPA and LTG (Myers et al., 2017). However, other patients with *PPP3CA*-related epilepsy showed no significant response to ASMs (Myers et al., 2017). Based on the data from patients in both the literature review and this study, epileptic spasms were controlled by ACTH in only one patient, while the others did not respond to ACTH. Additionally, none of the patients with epileptic spasms responded to VGB. This highlights the urgent need for targeted therapies for *PPP3CA*-related.

Genetic variations may induce functional alterations in proteins through modifications of distinct structural domains that govern specialized biological roles. These structural and functional distinctions suggest that pathogenic variants at different domain locations may manifest divergent detrimental consequences, ultimately contributing to heterogeneous phenotypic expressions (Liao et al., 2022). The molecular mechanisms underlying epileptogenesis associated with RD domain-truncating mutations remain insufficiently characterized. Notably, clinical observations reveal a distinct phenotypic spectrum among patients with RD domain mutations, characterized by markedly earlier epilepsy onset and polymorphic seizure manifestations compared to individuals harboring mutations in other functional regions. Furthermore, functional domain-specific phenotypic dichotomies demonstrate clear genotype-phenotype correlations: AID domain mutations induce multi-organ developmental anomalies in the absence of epileptic complications, while pathogenic variants consistently present as drug-resistant epilepsy syndromes refractory to conventional anticonvulsant therapies. This domain-dependent phenotypic specificity necessitates the establishment of dual research paradigms: (1) Domain-specific therapeutic targeting of ion channel dysregulation mechanisms and (2) Precision genome editing approaches including CRISPR-based mutation correction or antisense oligonucleotide-mediated transcript modulation. These research directions will fundamentally enhance both mechanistic comprehension and clinical management strategies for this genetically heterogeneous neurological disorder.

There are several strengths in this study. Firstly, we collected the largest single-center study to date. By leveraging this extensive patient samples, we combined the cases from this study with those described in the literature, focusing on detailed phenotypic descriptions. Secondly, this study demonstrated the genotype-phenotype correlation of *PPP3CA*-related epilepsy. We compared phenotypes across the CD, CaMB and RD and identified clinical phenotypes associated with different *PPP3CA* domains. Thirdly, this study identified seven novel variants and a gene inversion in *PPP3CA*-related DEE for the first time, expanding the genotypic spectrum of *PPP3CA*. Additionally, recurrent variants p.His92Arg, p.Asp234Glu, p.Glu282Lys, and p.Ser419Cysfs*33 were identified.

Admittedly, this study has several limitations. Firstly, the number of patients with *PPP3CA* variants is still relatively small, and more patients need to be enrolled for a more robust genotype-phenotype correlation analysis. Secondly, this is an observational study, and the observed results may be subject to confounding factors that we could not identify and control in this study. Thirdly, the follow-up period was not long enough to fully assess long-term outcomes.

## Conclusion

Over 60% of *PPP3CA* variants in patients with epilepsy were located in the RD. Variants located in the CD were exclusively missense variants. The majority of variants in the RD were truncating variants. Seizure onset in most cases of *PPP3CA*-related DEE occurred during infancy. The most common epileptic syndrome was IESS. Nearly 50% of patients with variants in the CD present with epileptic spasms associated with ASD. Most patients with *PPP3CA*-related epilepsy were drug-resistant. Additionally, our study reported a gene inversion in *PPP3CA*-related DEE for the first time. The variants p.His92Arg, p.Asp234Glu, p.Glu282Lys and p.Ser419Cysfs* 33 are the most frequently reported *PPP3CA* variants.

## Data Availability

The data presented in this study are available through Clinvar (https://www.ncbi.nlm.nih.gov/clinvar/), with the following accession numbers SCV006082273–SCV006082284. Further inquiries can be directed to the corresponding author.

## Footnotes

1
www.gnomad-sg.org/
