# Supplementary material for: New variants and genotype-phenotype correlation of PPP3CA-related developmental and epileptic encephalopathy
Source: Front Neurosci. 2025 Jun 6;19:1570997. doi: 10.3389/fnins.2025.1570997 (PMC12179222; doi:10.3389/fnins.2025.1570997)
Supplement: Supplementary file 1 [file Data_Sheet_1.docx]

The experimental data underlying this figure were generated by amplifying target genes with the primers listed below, followed by capillary electrophoresis on the Qsep100 system. The PCR conditions and primer specificity were validated through both in silico analysis (Primer-BLAST) and empirical optimization.

**Target gene primer design:**

- Retrieve the target gene's canonical mRNA and CCDS sequences from NCBI; cross-validate selected sequences against corresponding protein entries in UniProt.
- Perform primer design in SnapGene using the target vector's restriction sites; ensure matched primer melting temperatures (Tm) and screen for secondary structures (hairpins/dimers) using OligoAnalyzer to optimize PCR efficiency.
- test whether the primers can specifically amplify the target gene, in the Primer-BLAST tool provided on the NCBI website; input the designed primers and select the required species for retrieval.
- Send the verified primer sequence to the primer synthesis company for primer synthesis.
- The sequence of PCR primers was as follows,

PPP3CA-1F GCATTTCTGTTTTGTTGTCTGT,

PPP3CA-1R CATCATATACAAAAGTCAAACATAGCT;

PPP3CA-2F CACAGAAAAGAGTTGCATTTGTATT,

PPP3CA-2R TCACATCAACTGCTTATTTTAATGTC.

**Procedure**

- **Reaction Setup** (20–50 μL total volume):

Combine in a PCR tube:

- - - 2xTSINGKE ® Master Mix (Blue): 10 μL
    - Template DNA: 2 μL
    - Forward primer: 0.5 μL
    - Reverse primer: 0.5 μL
    - Nuclease-free water: 7 μL
    - Total volume: 20 μL
- **Thermal Cycling**:

| **Reaction** **temperature (℃)** | **Response time** | **Cycle-index** |
| --- | --- | --- |
| 95 | 2 min | 1X |
| 98 | 10s | 11X |
| 62 | 30s/-0.5℃ |  |
| 72 | 1 min |  |
| 98 | 10s | 24X |
| 56 | 30s |  |
| 72 | 1 min |  |
| 72 | 2 min | 1X |
| 4 | hold | - |
