# Supplementary material for: New variants and genotype-phenotype correlation of PPP3CA-related developmental and epileptic encephalopathy
Source: Front Neurosci. 2025 Jun 6;19:1570997. doi: 10.3389/fnins.2025.1570997 (PMC12179222; doi:10.3389/fnins.2025.1570997)
Supplement: Supplementary file 2 [file Data_Sheet_2.docx]

| **Table S1** Summary of the molecular and clinical findings of the 21 patients with *PPP3CA* pathogenic variants across eight studies [11-13, 24-28] | | | | | | | | | | |  |
| --- | --- | --- | --- | --- | --- | --- | --- | --- | --- | --- | --- |
|  | **Sex** | **Variants** | **Domain** | **Seizure** | | **developmental disability** | **Other clinical findings** | **EEG** | **Brain MRI** | **Diagnosis** | |
|  |  |  |  | **Age of onset** | **Seizure type** |  |  |  |  |  |  |
| 1 | M | c.275A>G/p.His92Arg | CD | 3m | ES, TS, MS | DD | hypotonia | Abnormal background, burst suppression in sleep, hypsarrhythmia | N | IESS, LGS | |
| 2 | F | c.275A>G/p.His92Arg | CD | 22m | ES | DD | hypotonia | Occipital polyspikeand-slow waves | N | IESS | |
| 3 | F | c.449A>T/p.Asn150Ile | CD | 8m | ES, TS, Ats, Ab | DD | Hypotonia,  ASD | Hypsarrhythmia | N | IESS, LGS | |
| 4 | F | c.702C>G/p.Asp234Glu | CD | 7m | ES, MS | DD | hypotonia,  ASD | Hypsarrhythmia | N | IESS | |
| 5 | F | c.760A>G/p.Arg254Gly | CD | 13y | GTCS | DD | hypotonia,  ASD | slow waves in the awake background with superimposed fast activities | N | DEE | |
| 6 | F | c.843C>G/p.His281Gln | CD | 3m | ES, | DD | hypotonia,  ASD | Hypsarrhythmia | mild generalized prominence of subarachnoid spaces | IESS | |
| 7 | F | c.844G>A/p.Glu282Lys | CD | 4y | ES, FS | DD | hypotonia,  ASD | Abnormal background frequent GSW, PS and wave | mild cortical sulcal prominence | DEE | |
| 8 | F | c.1177A>T p.Lys393* | CaMB | 4y | FS, AA, GTS | DD | N | GSw, SW complexes on bilateral frontotemporal regions | N | DEE | |
| 9 | F | c.1238T>C p.Leu412Pro | CaMB | 3y | FS, MS, AA, GTS | DD | hypotonia | Fronto-temporal SW - TA or GLv | N | Hypotonia | |
| 10 | F | c.1255_1256delAG/p.Ser419Cysfs∗31 | RD | 2m | GTCS | DD | N | Sharp, spike, sharp-slow, spike-slow wave | brain dysplasia, thin corpus callosum, and widened brain interval | DEE | |
| 11 | F | c.1255_1256delAG/p.Ser419Cysfs∗31 | RD | 1day | ES | DD | Generalized hypotonia | hypsarrhythmia | Normal | IESS | |
| 12 | M | c.1255_1256delAG/p.Ser419Cysfs∗31 | RD | 3m | ES, GTS, FS | DD | hypotonia | Multifocal IEDs, burst suppression-like | Thin corpus callosum | IESS | |
| 13 | M | c.1258_1259insAGTG /p.Val420Glufs 32 | RD | 2m | ES, MS, Ats | DD | hypotonia | hypsarrhythmia | broadened bilateral frontotemporal extracerebral space | IESS | |
| 14 | F | c.1283insC/p.Thr429Asnfs*22 | RD | 13m | ES, MS, | DD | hypotonia | Hypsarrhythmia, multifocal discharge and spasms | N | IESS | |
| 15 | M | c.1283insC/p.Thr429Asnfs*22 | RD | 2y | ES, Ats, GTCS, MS | DD | N | Slow background waves, PS, GSW, hypsarrhythmia | N | IESS | |
| 16 | F | c.1290dupC/p.Met431Hisfs*20 | RD | 6m | ES, FS | DD | N | Hypsarrhythmia | N | IESS | |
| 17 | M | c.1299dupC/p.Ser434Glnfs*17 | RD | 4m | ES, GTCS | DD | hypotonia | Generalized interictal discharges consistent with hypsarrhythmia | prominent pericerebral spaces and asymmetric ventricles | IESS | |
| 18 | M | c.1308_1311dupACTT/p.Ser438Thrfs*14 | RD | 18m | GTCS, MS | DD | hypotonia, ASD | multifocal epileptic discharges | N | DEE | |
| 19 | M | c.1324C>T/p.Gln442* | RD | 2m | GTCS, ES, MS | DD | receding mandible and narrow palate, atrial septal defect | Sharp waves and spikes, sleep spindles | Ventricular dilation, supratentorial brain atrophy, thinned corpus callosum | IESS | |
| 20 | M | c.1333C>T/p.Gln445* | RD | 1.5m | ES, TS, FS, MS, clonic | DD | hypotonia | Hypsarrhythmia, MFD, GSW | Delayed myelination, T2 hyperintensity, white matter loss | IESS | |
| 21 | F | c.1339G>A/p.Ala447Thr | RD | 3.5y | GTCS, TS, ES | DD | hypotonia | MFD, hypsarrhythmia in sleep, frontal sharp and spikes | Mild prominence of subarachnoid spaces | DEE | |
| **Note:** M male, F female, d days, m months, y years, RD Regulatory domain, CD catalytic domain, Ats atypical absence seizures, ES epileptic spasm, MS myoclonic seizure, FS focal seizure, GTCS generalized tonic–clonic seizure, TS tonic seizure, IESS infantile epileptic spasms syndrome, DEE developmental and epileptic encephalopathy, DD developmental delay;  EEG electroencephalography, MRI magnetic resonance imaging, N normal | | | | | | | | | | |  |

| **Table S2** The ACMG criteria and classification of *PPP3CA* variations in our study | | | | | |
| --- | --- | --- | --- | --- | --- |
|  | **Variants** | **Inheritance** | **Location** | **Categorization** | **ACMG criteria** |
| 1 | c.275A>G/p.His92Arg | de novo | CD | Likely pathogenic | PS2+PM2+PP3 |
| 2 | c.275A>G/p.His92Arg | de novo | CD | Likely pathogenic | PS2+PM2_Supporting+PP3 |
| 3 | c.702C>A/p.Asp234Glu | de novo | CD | Pathogenic | PS2+PM2+PP2+PP3 |
| 4 | c.702C>A/p.Asp234Glu | de novo | CD | Likely pathogenic | PS2+PM2_Supporting+PP2+PP3 |
| 5 | c.1255_1256delAG/ p.Ser419Cysfs*31 | de novo | RD | Pathogenic | PVS1+PS2+PM2 |
| 6 | c.1284_1287dup/ p.Gly430Asnfs*22 | de novo | RD | Pathogenic | PVS1+PS2+PM2 |
| 7 | c.1336_1337insCAAA/p.Ser446Thrfs*6 | de novo | RD | Pathogenic | PVS1+PS2+PM2 |
| 8 | inversion between intron 11-13 | de novo | RD | Pathogenic | PVS1+PS2+PM2 |
| 9 | c.1338dup/p.Ala447fs* 4 | de novo | RD | Pathogenic | PVS1+PS2+PM2 |
| 10 | c.1258_1259 insAGTG / p.Vla420Glufs*32 | de novo | RD | Pathogenic | PVS1+PS2+PM2 |
| 11 | c.1283_1284insC/ p.Thr429Asnfs*22 | de novo | RD | Pathogenic | PVS1+PS2+PM2 |
| 12 | c.1311_1312insACTT/p.Ser438fs*14 | de novo | RD | Pathogenic | PVS1+PS2+PM2 |
| 13 | c.1354_1356delinsCAATA/p.Ile452Glnfs*2 | de novo | RD | Pathogenic | PVS1_Strong+PS2+PM2 |
| 14 | c.375A>T/p.Lys459* | de novo | RD | Likely pathogenic | PS2_Moderate+PVS1_Strong+PM2_Supporting |
| 15 | c.1251_1252del/p.Ser417Argfs*33 | de novo | RD | Pathogenic | PVS1+PS2_Moderate++PM2_Supporting |

Note: PVS1, Pathogenic Very Strong 1; PS2, Pathogenic Strong 2; PM2, Pathogenic Moderate 2; PP2, Pathogenic Supporting 2; PP3, Pathogenic Supporting 3

**Table S3** Genetic characteristics of *PPP3CA* missense variants in this study

| **Variants** | **SIFT** | **PP2_**  **HDIV** | **Mutation Taster** | **M_CAP** | **CADD** | **DANN** | **FATHMM_MKL** | **GERP** | **SiPhy** |
| --- | --- | --- | --- | --- | --- | --- | --- | --- | --- |
| c.275A>G/  p. His92Arg | D (0.000) | PD (1.000) | DC (1.000) | D (0.128) | D (26.2) | D (0.997) | D (0.984) | C (5.07) | C (15.162) |
| c.702C>A/  p. Asp234Glu | D (0.000) | PD (0.999) | DC (1.000) | D (0.059) | D (27) | D (0.997) | D (0.94) | C (5.26) | C (9.325) |

Note: C, conserved; CADD, Combined Annotation Dependent Depletion; D, damaging; DC, disease-causing; DANN, Domain Adversarial Neural Networks; Fathmm-MKL, Functional Analysis through Hidden Markov Models–Multiple Kernels Learning; GERP, Genomic Evolutionary Rate Profiling; M_CAP, Mendelian Clinically Applicable Pathogenicity; P, pathogenic; PD, probably damaging; PP2_HDIV, polyphen2_HDIV; SIFT, Sorting Intolerant from Tolerant.
