# Supplementary material for: New variants and genotype-phenotype correlation of PPP3CA-related developmental and epileptic encephalopathy
Source: Front Neurosci. 2025 Jun 6;19:1570997. doi: 10.3389/fnins.2025.1570997 (PMC12179222; doi:10.3389/fnins.2025.1570997)
Supplement: Supplementary file 3 [file Data_Sheet_3.docx]

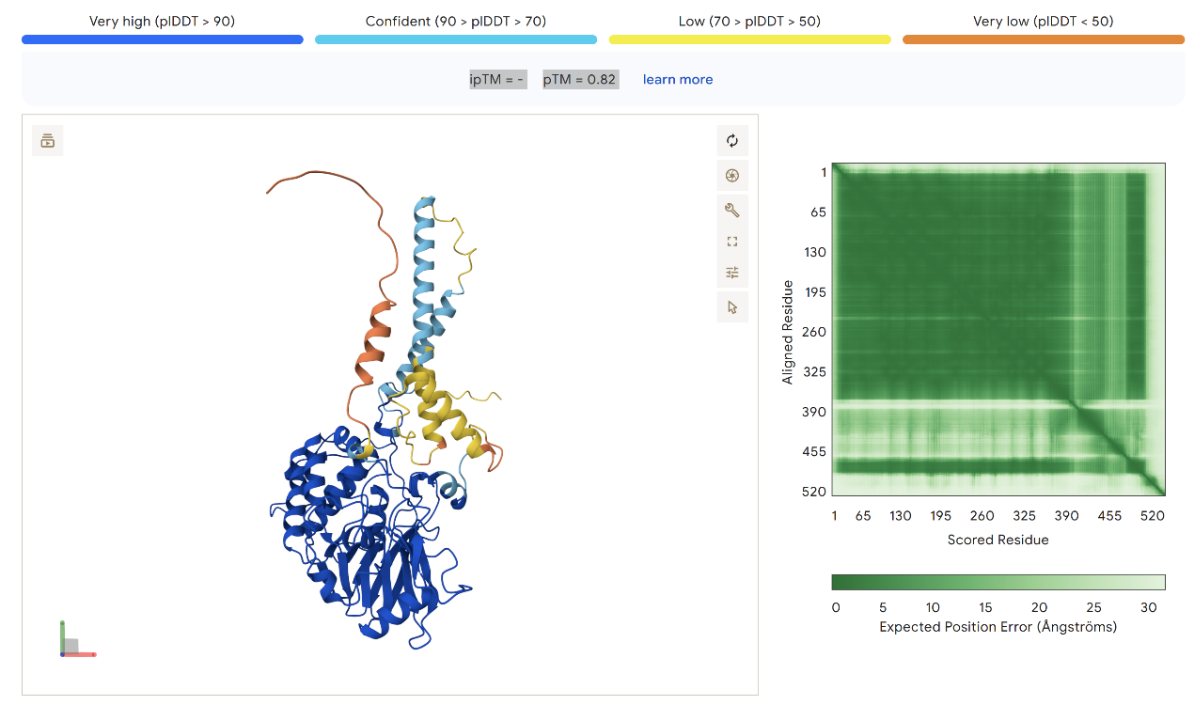


**Fig. S1** AlphaFold2 prediction image (pTM=0.82).


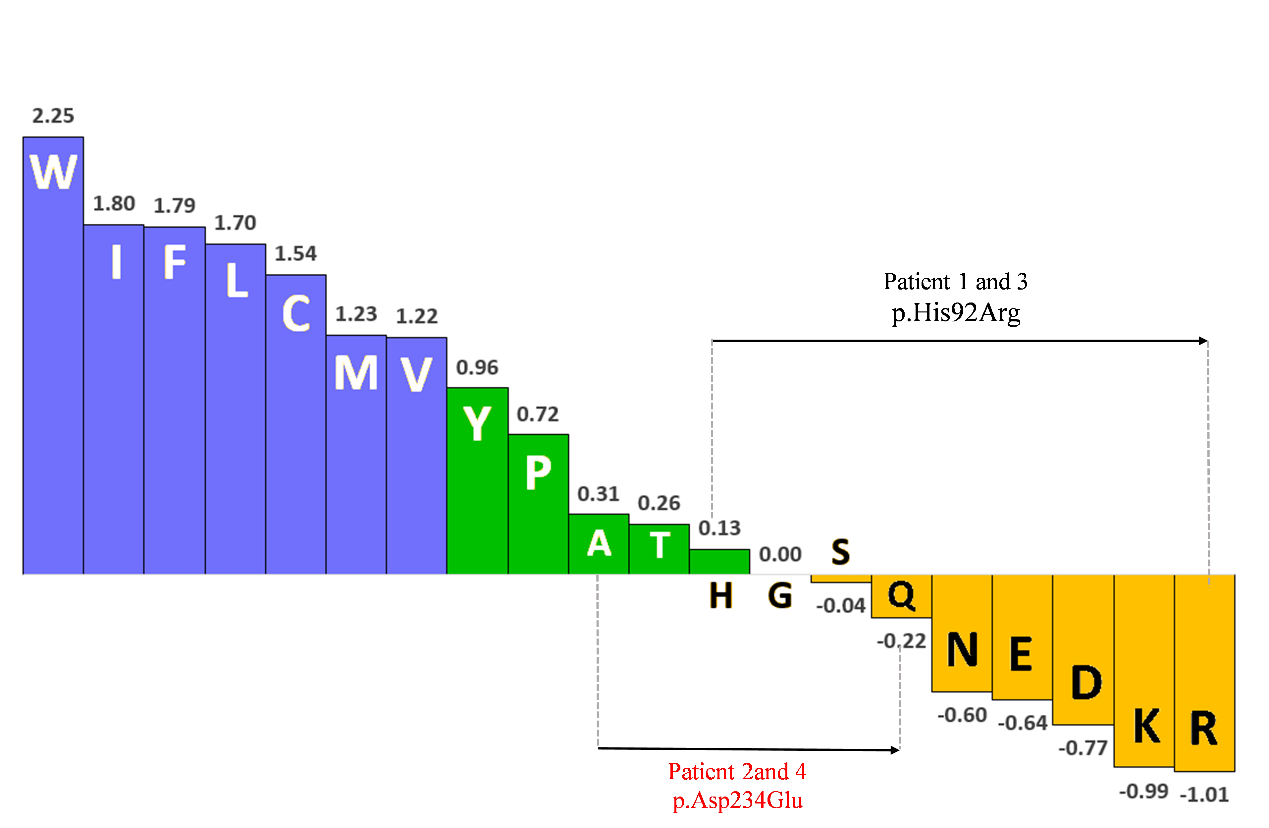


**Fig.S2** Variants with obvious changes in DDGs are highlighted in red. Hydrophobicity of amino acids calculated by the Fauch`ere and Pliska hydrophobicity scale. The amino acids shown in blue are hydrophobic, those in green are neutral, and those in yellow are hydrophilic. The hydrophobicity gradually decreased from left to right. Amino acids with high positive values are more hydrophobic, whereas amino acids with low negative values are more hydrophilic.
